# GallOnt: An ontology for plant gall phenotypes

**DOI:** 10.3897/BDJ.12.e128585

**Published:** 2024-08-26

**Authors:** Andrew R Deans, Louis Frank Nastasi, Charles Davis

**Affiliations:** 1 Frost Entomological Museum, The Pennsylvania State University, University Park, United States of America Frost Entomological Museum, The Pennsylvania State University University Park United States of America

**Keywords:** phenotype, plants, zoocecidium, morphology, cecidology, Cecidomyiidae, Cynipidae, Diplolepididae, Cynipoidea

## Abstract

Galls are novel plant structures that develop in response to select biotic stressors. These structures, extended phenotypes of the inducer, usually serve to protect and feed the inducer or its progeny. This life history strategy has evolved dozens of times, and tens of thousands of species — including many bacteria, fungi, nematodes, mites and insects — are capable of manipulating plants in this way. The variation in gall phenotypes is extraordinary across species but usually predictable for each species of inducer. We introduce here a new ontology, GallOnt, that facilitates consistent descriptions and the semantic representation of and reasoning over plant gall phenotype data. GallOnt was largely developed from ontologies in the Open Biological and Biomedical Ontology (OBO) Foundry and stands to connect plant gall phenotypes to knowledge derived from model plant systems, including genotype-phenotype and agricultural research. We also introduce the idea of a new gall data standard — Minimum Information for the Description of Galls (MIDG version 0.1) — as a starting point for discussions regarding cecidology best practices.

## Introduction

**Figure 1.**
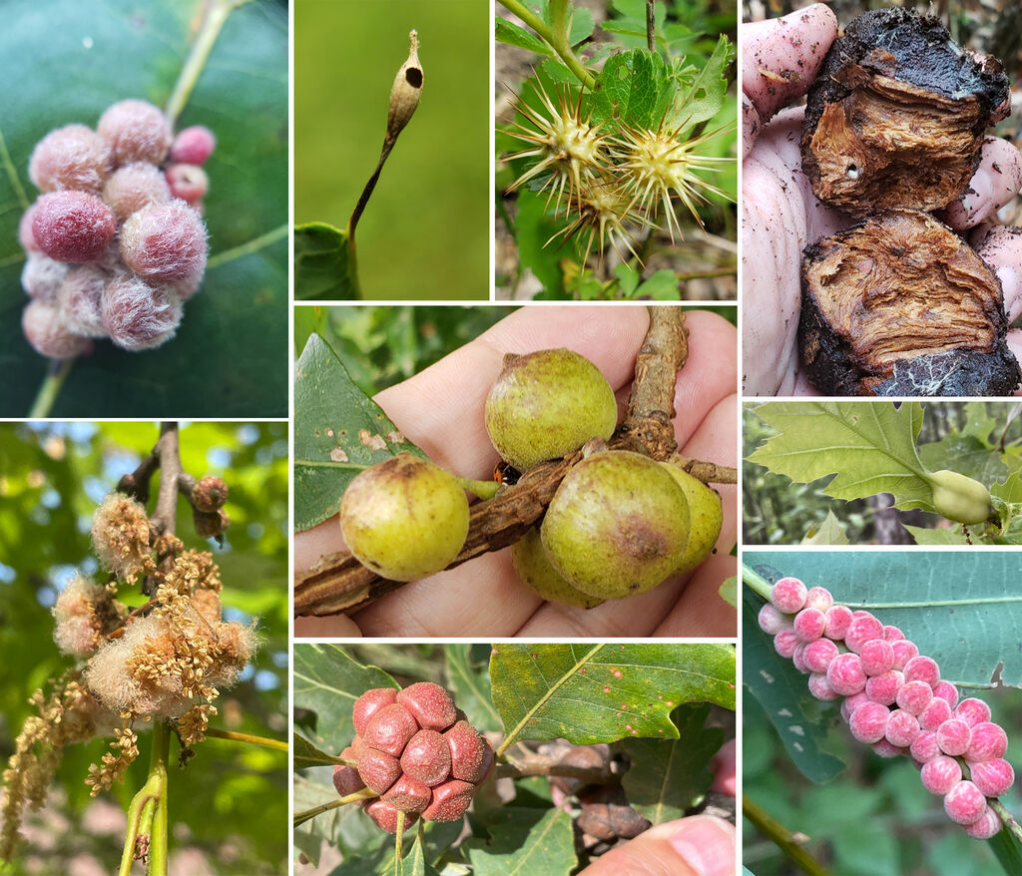
Some of the galls formed by plants in response to manipulation by gall wasps (Hymenoptera, Cynipoidea). Clockwise from the spiny gall at the top: *Diplolepisbicolor* leaf galls on *Rosa* sp.; *Holocynipsmaxima* "root" gall on *Quercusmontana*; *Melikaiellatumifica* mid-rib gall on *Quercusrubra*; undescribed mid-rib gall cluster on *Quercusmontana*; *Andricusquercusstrobilanus* bud gall cluster on *Quercusbicolor*; *Callirhytisquercusoperator* catkin gall on *Quercusrubra*; *Callirhytispiperoideas* mid-rib gall cluster on *Quercusvelutina*; *Andricuschinquapin* stalked leaf gall on *Quercusalba*; (middle) *Disholcaspisquercusmamma* gall cluster on *Quercusbicolor* stem. All photos (CC BY 4.0) by Andrew R. Deans.

The ability to induce galls on plants has evolved independently many times across the phylogeny of Life, with evidence of this kind of interaction dating back at least to the Middle Devonian (385 million years ago; Labandeira (2021)). Despite a long evolutionary history, the prevalence of this phenomenon in nature (Fig. 1), and broad research interest, progress towards understanding the physiological mechanisms of gall induction has been incremental ([Bibr B11499038][Bibr B11499024], Hearn et al. 2019). However, interest in researching the mechanisms of gall induction and development remains high, and general interest in gall biology is surging. Research-grade records of gall inducers in iNaturalist, for example, are growing exponentially (see GBIF occurrences for iNaturalist records of gall-inducing wasps in the superfamily Cynipoidea; https://doi.org/10.15468/dl.e4wud2), and a new resource has been established to facilitate the identification of gall-inducers in North America (Gallformers Contributors 2024).

We provide here another resource, a structured glossary of gall phenotype terms, that facilitates natural language and semantic descriptions of gall forms and can forge connections to knowledge generated through model organism research. As proposed in other systems (Deans et al. 2015), genotype-phenotype data from plant models, may shed light on the mechanisms of gall induction and the subsequent, predicable morphology we observe in the extended phenotypes of gall inducers. The logic inherent in this ontology also allows for computation across phenotype datasets, which may reveal hidden patterns in gall traits across a broad array of gall-inducing species. Similar approaches are used already in taxonomy (Balhoff et al. 2013) and evolutionary developmental biology (Edmunds et al. 2015).

Through this paper, we aim to announce this new ontology, which was developed using Open Biological and Biomedical Ontology (OBO) Foundary principles (Jackson et al. 2021) and with community input; demonstrate its utility as a controlled vocabulary and as a computational tool; and provide some examples of its use in describing and referring to gall phenotypes.

## Methods

The development process largely transitioned through three main phases: (1) glossary development, i.e. gathering relevant concepts and terms, with community input; (2) structuring the glossary as an ontology, using established best practices; and (3) demonstrating the computational nature of the ontology, by reasoning over phenotype data.

### Glossary development

Given the modest size of the lexicon used to describe gall morphology, we opted for a fully manual assembly of the ontology. We extracted gall terms from original descriptions catalogued by Nastasi and Deans (2021) and from two monographs on insect galls, by Felt (1940), Weld (1959). The descriptive terms were then manually matched to classes in OBO Foundry ontologies (Jackson et al. 2021), mainly the Phenotype and Trait Ontology (PATO; Gkoutos et al. (2005)) and the Plant Ontology (PO; Cooper and Jaiswal (2016)). Terms that did not match any classes in existing ontologies were given Aristotelian definitions (Smith 2023), in line with OBO Foundry principles. Given that galls are plant structures, we relied primarily on botanical references as sources for new definitions (e.g. Beentje (2016)). The resulting "gall glossary" was availed as a spreadsheet (Deans et al. 2021), with the following data columns and shared with the broader community of gall biologists for feedback:

- term (the label for the concept);
- definition (human readable genus differentia);
- category (parent class);
- URI (link to a matching class in an existing ontology, like PATO or the PO);
- relatedURI (link to a source if the term is not in an ontology already, or class that is close to the gall term or a class that is the parent of newly-defined term);
- exactSynonyms (labels with identical meaning);
- partialSynonyms (labels that are closely related, but not exact synonyms);
- notes (free text field for curator notes);
- example (links to occurrences in iNaturalist that illustrate each term).

Community recommendations were incorporated into the ontology (see next section) or set aside for future consideration (i.e. documented as issues in the GitHub repository; https://github.com/adeans/gallont/issues).

### Ontology development

We used the Ontology Development Kit (ODK) tool set and protocol (Matentzoglu et al. 2022) to formalise the gall glossary as a standalone ontology. Following extensible ontology development (XOD) principles (He et al. 2018) and guidance provided by Vasilevsky et al. (2022), we composed the the Plant Gall Ontology (GallOnt) largely by adopting classes and properties from other ontologies in the OBO Foundry. This paper serves, in part, to help satisfy the minimum reporting requirements for an ontology (MIRO; Matentzoglu et al. (2018)), specifically the elements related to knowledge acquisition and quality assurance. GallOnt was approved by the OBO Operations Committee in March 2024 and availed through the OBO Foundry. Versioning is managed in our GitHub repository: https://github.com/adeans/gallont. In line with ontology development best practices, we include the following metadata for each new class: label (rdfs:label), definition, date created (dcterms:date, using ISO-8601), contributor (dcterms:creator, as an ORCID), source reference if applicable (dcterms:source, usually as a DOI), alternative labels (i.e. synonyms; https://purl.obolibrary.org/obo/IAO_0000118). We also attempted to add links to occurrences in iNaturalist that exemplified each class (in seeAlso).

### Reasoning

**Figure 2.**
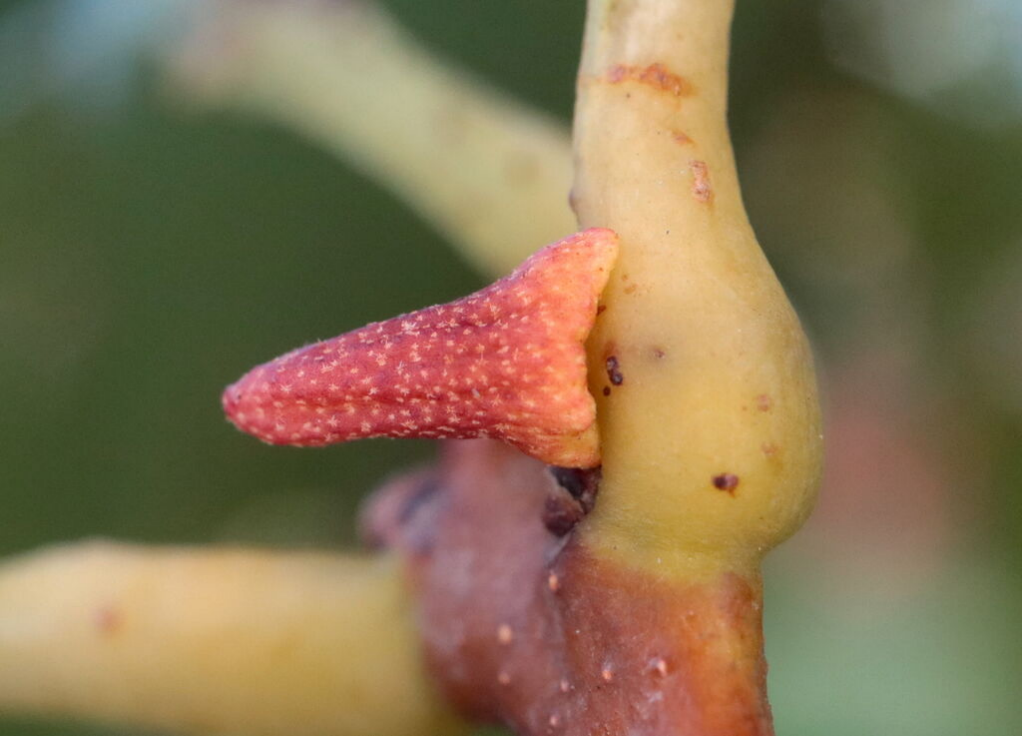
*Zopheroterascuneatum* gall on white oak (*Quercusalba*), from an observation in iNaturalist (https://www.inaturalist.org/observations/96124447) by Jeff Clark (CC BY 4.0)

We extracted gall phenotypes from natural language descriptions of galls that are induced on white oak, *Quercusalba* Linnaeus, 1753, as listed at the Gallformers website (https://gallformers.org). For example, Weld (1944) described the gall of *Zopheroterascuneatum* Weld, 1944 (Fig. 2) as: "Conical, red, 3.0–4.4 mm long by 1.5 mm broad at base, attached to the very base of the petiole in fall just as the leaves are turning. On young trees or sprouts from stumps". We parsed that description into six characters, which we translated into semantic phenotypes that were applied to a mock specimen (individual) for that species. Table 1 provides examples of how descriptive data were translated into semantic statements. We attempted to translate each natural language phrase into semantic statements in Protégé (version 5.6.3; Musen (2015)), following protocols outlined by Balhoff et al. (2013) and Mikó et al. (2014). The resulting dataset was then queried using descriptive logic (DL), with the ELK reasoner (version 0.4.3; Kazakov et al. (2012)) implemented in Protégé. Suppl. material 2 lists all the natural language phenotypes we attempted to capture, with links to original sources of those phenotype descriptions.

## Results

The final version of the gall glossary yielded 136 concepts (classes) that are directly relevant to gall morphology. We assigned each class a primary label, sometimes called a "preferred term" (Yoder et al. 2010) and matched an additional 56 synonymous terms to their respective classes. For example, some galls have multiple chambers within them, each of which contains a larva of the inducer. We refer to this type of gall as "polythalamous" (the primary label) and define this phenotype as "an internal gall trait in which more than one larval chamber is present for the inducer species" (the Aristotelian definition). Synonyms (secondary labels) assigned to this class include "multichambered", "multilocular" and "multi-celled".

The size and complexity of the resulting ontology, version 2024-04-19, is reported in Table 2. Of the 394 classes, 53 are unique to GallOnt. The remaining 341 were derived from other ontologies, including 161 from PATO, 120 from PO, 31 from NCBITaxon, 14 from BFO, six from GO, three from CARO and one each from FLOPO, IAO, ENVO, OBI, UBERON and NCI_Thesaurus. GallOnt has 132 object properties, three of which originate in GallOnt: *induced_by* (to designate the organism responsible for inducing the gall), *has_seasonal_maturity* and *has_seasonal_emergence* (to represent seasonal elements of plant gall life cycles). Remaining properties are from the Relation Ontology (RO; Smith et al. (2005)). The ontology is available in three formats through the OBO Foundry (https://obofoundry.org/ontology/gallont.html): OWL, OBO and JSON.

The reasoning dataset was created as a separate test ontology (see Suppl. material 1), to which we added 72 individual galls and gall clusters, each with an average of about nine phenotypes (x̄ = 8.96; 645 total for the dataset) and one measurement (x̄ = 0.93; 67 total data property assertions for the dataset). We formulated several questions a biologist could ask, regarding galls on *Quercus*, as recorded in this dataset and generated DL queries for each. The results are presented in Table 3.

## Implementation, caveats and future directions

We demonstrate above and in the supplementary files how this ontology can be used to compose semantic phenotypes for plant galls. The ontology also stands as a controlled vocabulary for describing gall morphology. We recommend using a character:character state or entity:quality format, for example in a spreadsheet or as rendered in many contemporary taxonomic descriptions (e.g. see species descriptions in Mikó et al. (2014)). This syntax is intuitive for human readers and lends itself to further processing, for example, to generate semantic statements using a script (Tarasov et al. 2022). As described above, we include human readable, logical definitions in the ontology, as well as links to exemplar galls in iNaturalist, for most of the classes, to aid in understanding each concept. One can browse the ontology directly by loading the OWL file in Protégé or through several online ontology sites (e.g. EMBL-EBI Ontology Lookup Service (OLS), Ontobee and BioPortal).

Our small survey of gall descriptions also revealed inconsistencies and incompleteness of the phenotypes that were represented, making it difficult to confidently query published knowledge of galls. For example, only 33 descriptions out of 67 reported whether the gall was mono- or polythalamous (highlighted rows in Table 3). As a starting point for future conversations, we propose that cecidologists consider including the following minimum information for the description of galls (MIDG version 0.1), in future publications:

- host plant species
- location of gall on the plant (part of <some plant anatomical entity>);
- maximum diameter in mm (measured in the axis that is perpendicular to the axis of attachment to the plant);
- maximum height in mm, from point of attachment to the plant or length in mm, if describing a non-deciduous gall (i.e. measured along the axis of the plant part affected);
- internal qualities, including
  - whether the gall is mono- or polythalamous (for galls induced by Cynipoidea or Cecidomyiidae)
  - whether the gall is solid, hollow, spongy, succulent etc.;
- date of appearance on the plant if known;
- date of emergence of the inducer if known;
- surface texture;
- shape;
- colour pattern;
- shedability;
- spatial pattern (confluent, clustered, scattered or solitary).

GallOnt likely is not yet sufficient for describing every type of gall nor all the complex phenotypes exhibited in these structures. For example, the ontology does not yet include many classes needed to represent histological traits (tissue qualities) nor temporal changes in gall morphology (i.e. stages of gall development). During the initial development, we also focused almost exclusively on phenotypes expressed in galls found in North America, as induced by cynipoid wasps. Expanding the ontology to cover galls induced by thrips (Thysanoptera), mites (especially Eriophyidae), gall midges and other flies (Diptera) and non-arthropod inducers, in other parts of the world, will undoubtedly require additional classes and new versions of MIDG. New classes and other modifications to the ontology can be proposed using the GitHub issue tracker linked above and ongoing development in relevant ontologies (see below) will likely facilitate more sophisticated representation of gall phenotypes.

GallOnt can be used in generating Gene Ontology annotation files (GAFs; The Gene Ontology Consortium et al. (2023)), when reporting on gene expression studies of galls (for example, Hearn et al. (2019)Martinson et al. (2022)). We are actively developing sections of the Plant Trait Ontology (TO) and Plant Stress Ontology (PSO) (Cooper et al. 2018) that refer to gall development and inducer-plant interactions, to more fully integrate plant gall concepts with tools used for discovery in model plant systems. Relatedly, GallOnt is also available in AgroPortal (https://agroportal.lirmm.fr/ontologies/GALLONT; Jonquet et al. (2018)), to facilitate gall research related to agriculture. Several species of gall-inducers are important pests, including *Dryocosmuskuriphilus* Yasumatsu, 1951 (Cynipidae, Cynipini) on sweet chestnut trees (Fagaceae, *Castaneasativa* Mill.), Hessian fly (*Cecidomyiadestructor* Say, 1817) on cereals and many related gall midges (Diptera, Cecidomyiidae; see volumes by Horace Francis Barnes (1902–1960), starting with Barnes (1946)). Other species of gall inducer, especially *Agrobacterium* spp. (Alphaproteobacteria, Rhizobiaceae), have application in transforming plants (Nester 2015). These research areas could benefit from future refinements to GallOnt.

## Conclusions

We provide here the first ontology designed to represent plant gall phenotypes. The ontology was developed with the latest tools, to be extensible, accessible and persistent, and we demonstrate its utility as a reasoning tool. We invite potential users and contributors to view the documentation at the GallOnt site (https://adeans.github.io/gallont/), to provide new terms and other refinements and to offer additional use cases.

## Supplementary Material

509270B6-099D-5DC5-98B8-2889BDF5BDD1 10.3897/BDJ.12.e128585.suppl1 Supplementary material 1 GallOnt reasoning test ontology Data type Web Ontology Language (OWL) file Brief description This file contains the plant gall ontology and numerous individuals, each of which represents a gall on white oak (Quercussect.Quercus, mostly on *Q.alba*). This file was used to demonstrate reasoning over phenotype data, using descriptive language (DL) queries, as implemented with ELK reasoner (version 0.4.3) in the software application Protégé (version 5.6.3). File: oo_1054529.owl https://binary.pensoft.net/file/1054529 Andrew R. Deans

6CB8A42C-0EB7-5348-8451-2A55ABF5DE85 10.3897/BDJ.12.e128585.suppl2 Supplementary material 2 White oak gall phenotypes Data type morphological characters Brief description This spreadsheet contains natural language descriptions of all described galls on white oak (*Quercusalba*). The descriptions are parsed into individual characters and links (URIs) are provided for each source. File: oo_1057050.xlsx https://binary.pensoft.net/file/1057050 Andrew R. Deans

## Figures and Tables

**Table 1.** Examples of gall traits of *Zopheroterascuneatum* Weld, 1944 as originally described by the author (column 1), translated into semantic statements that are composed from multiple ontologies (column 2). Weld (1944) describes the spectrum of phenotypes he observed (column 1), but we are generating an example dataset of instances, in this case, mock specimens. See notes (column 3) for explanation.

original description	phenotype	notes
red	'has quality' some red	
3.0–4.4 mm long	hasHeightInMm 4.4	We are generating a mock dataset comprised of individuals; therefore, we chose to represent just the maximum value from the range, for the "specimen" that represents this taxon.
attached to the very base of the petiole	'part of' some petiole	
in fall	'has seasonal maturity' value autumn	'has seasonal maturity' is an object property created for GallOnt
conical	'has quality' some conical	
1.5 mm broad at base	hasDiameterInMm 1.5	hasDiameterInMm and hasHeightInMm are data properties created for GallOnt
Host.—*Quercusalba*	'has host' some 'Quercus alba'	Taxon names mostly come from the NCBI Taxonomy
	'host of' some 'Zopheroterascuneatum (agamic)'	'Zopheroterascuneatum (agamic)' was created as a child of Zopheroterascuneatum and designated as an asexual organism
	'plant gall'	to declare that this is a gall

**Table 2.** GallOnt ontology metrics.

Axiom	6,042
Logical axiom count	973
Declaration axioms count	666
Class count	394
Object property count	132
Data property count	3
Individual count	13
Annotation Property count	128

**Table 3.** Descriptive Logic (DL) Queries. Examples of some biological questions that could be answered through queries of semantic plant gall phenotype data. Rows highlighted in yellow reveal the incompleteness of the original descriptions, in that there are 67 galls in the dataset, but only 33 have data regarding the number of larvae inside (i.e. whether they are mono- vs. polythalamous). *Note that the results in Protégé yield counts and also every individual that matches the query; only counts are listed here. **ELK does not support queries of data properties, but a query using another reasoner would return results; unfortunately, our ontology is too complex for the other reasoners in Protégé at this time.

Question	DL Query	Result*
How many plant galls are in this dataset?	'plant gall'	67
How many of these galls occur on *Quercusalba*?	'plant gall' and 'has host' some 'Quercusalba'	65
How many galls occur on an inflorescence?	'plant gall' and 'part of' some inflorescence	1
How many galls occur on stems and branches?	'plant gall' and 'part of' some 'shoot axis'	15
How many galls occur on leaves?	'plant gall' and 'part of' some leaf	30
How many galls occur on leaf veins?	'plant gall' and 'part of' some 'leaf vein'	9
How many galls occur on leaf mid-veins?	'plant gall' and 'part of' some 'leaf midvein'	3
How many galls occur on the roots?	'plant gall' and 'part of' some 'root system'	1
How many galls fall to the ground when mature?	'plant gall' and 'has quality' some 'deciduous (generic)'	13
How many galls are fully integrated into plant tissue?	'plant gall' and 'has quality' some non-deciduous	7
How many galls have a kapéllo?	'plant gall' and 'has part' some kapéllo	0
How many galls are monothalamous?	'plant gall' and 'has quality' some monothalamous	22
How many galls are polythalamous?	'plant gall' and 'has quality' some polythalamous	11
How many galls mature in autumn and have an asexual occupant?	'has seasonal maturity' value autumn and 'host of' some 'asexual organism'	16
How many galls mature in autumn and have a sexual occupant?	'has seasonal maturity' value autumn and 'host of' some 'gonochoristic organism'	0
How many galls are induced on buds by asexual organisms?	'induced by' some 'asexual organism' and ('part of' some bud)	4
How many galls are induced on buds by sexual organisms?	'induced by' some 'gonochoristic organism' and ('part of' some bud)	0
How many galls have a diameter greater than 5 mm?	'plant gall' and hasDiameterInMm some xsd:integer [>5]	19**
